# Supplementary material for: Large scale prospective evaluation of co-folding across 557 Mac1-ligand complexes and three virtual screens
Source: bioRxiv. 2025 Dec 29:2025.12.25.696505. Preprint. [Version 2] doi: 10.64898/2025.12.25.696505 (PMC12776374; doi:10.64898/2025.12.25.696505)
Supplement: Supplement 1 — • Supplementary Table6 : PDB accessions by cutoff date [file media-1.pdf]

Supplementary Table6: Protein-ligand structure complexes deposited in PDB prior to training dates (AF3, Chai-1 and Boltz-2).

Target	PDB ID (deposition before AF3, Chai-1 training dates: 2021-09-30)	PDB ID (additional deposition before Boltz-2 training date: 2023-06-01)
**Mac1**	7VLO, 7FAC, 7EO8, 7END, 7LMH, 7LMJ, 7LG7, 7LCP, 7LCQ, 7BF3, 7BF4, 7BF5, 7KQP, 5S18, 5S1A, 5S1C, 5S1E, 5S1G, 5S1I, 5S1K, 5S1M, 5S1O, 5S1Q, 5S1S, 5S1U, 5S1W, 5S1Y, 5S20, 5S22, 5S24, 5S26, 5S27, 5S28, 5S29, 5S2A, 5S2B, 5S2C, 5S2D, 5S2E, 5S2F, 5S2G, 5S2H, 5S2I, 5S2J, 5S2K, 5S2L, 5S2M, 5S2N, 5S2O, 5S2P, 5S2Q, 5S2R, 5S2S, 5S2T, 5S2U, 5S2V, 5S2W, 5S2X, 5S2Y, 5S2Z, 5S30, 5S31, 5S32, 5S33, 5S34, 5S35, 5S36, 5S37, 5S38, 5S3A, 5S3B, 5S3C, 5S3D, 5S3E, 5S3G, 5S3H, 5S3I, 5S3J, 5S3K, 5S3L, 5S3M, 5S3N, 5S3O, 5S3P, 5S3Q, 5S3R, 5S3S, 5S3T, 5S3U, 5S3V, 5S3W, 5S3X, 5S3Y, 5S3Z, 5S40, 5S41, 5S42, 5S43, 5S44, 5S45, 5S46, 5S47, 5S48, 5S4A, 5S4B, 5S4C, 5S4F, 5S4H, 5S4I, 5S4J, 5S4K, 7KG6, 7KG7, 7KG0, 7KFP, 5RVJ, 5RVK, 5RVL, 5RVM, 5RVN, 5RVO, 5RVP, 5RVQ, 5RVR, 5RVS, 5RVT, 5RVU, 5RVV, 5RS7, 5RS8, 5RS9, 5RSB, 5RSC, 5RSD, 5RSE, 5RSF, 5RSG, 5RSH, 5RSI, 5RSJ, 5RSK, 5RSL, 5RSM, 5RSN, 5RSO, 5RSP, 5RSQ, 5RSR, 5RSS, 5RST, 5RSU, 5RSV, 5RSW, 5RSX, 5RSY, 5RSZ, 5RT0, 5RT1, 5RT2, 5RT3, 5RT4, 5RT5, 5RT6, 5RT7, 5RT8, 5RT9, 5RTA, 5RTB, 5RTC, 5RTD, 5RTE, 5RTF, 5RTG, 5RTH, 5RTI, 5RTJ, 5RTK, 5RTL, 5RTM, 5RTN, 5RTO, 5RTP, 5RTQ, 5RTR, 5RTS, 5RTT, 5RTU, 5RTV, 5RTW, 5RTX, 5RTY, 5RTZ, 5RU0, 5RU1, 5RU2, 5RU3, 5RU4, 5RU5, 5RU6, 5RU7, 5RU8, 5RU9, 5RUA, 5RUC, 5RUD, 5RUE, 5RUF, 5RUG, 5RUH, 5RUI, 5RUJ, 5RUK, 5RUL, 5RUM, 5RUN, 5RUO, 5RUP, 5RUQ, 5RUR, 5RUS, 5RUT, 5RUU, 5RUV, 5RUW, 5RUX, 5RUY, 5RUZ, 5RV0, 5RV1, 5RV2, 5RV3, 5RV4, 5RV5, 5RV6, 5RV7, 5RV8, 5RV9, 5RVA, 5RVB, 5RVC, 5RVD, 5RVE, 5RVF, 5RVG, 5RVH, 5RVI, 7K0G, 7K0H, 6XHL, 6Z72, 6Z6I, 6YWL, 6W8Q, 6W8Y, 6W8Z, 6W91, 6W8K, 6W8M, 6W7H, 6W0T, 6VUQ, 6LNY, 6LO0, 6LNQ	8SH8, 8GIA, 8HUS, 8C1A, 7FR0, 7FR1, 7FR2, 7FR3, 7FR4, 7FR5, 7FR6, 7FR7, 7FR8, 7FR9, 7FRA, 7FRB, 7FRC, 7FRD, 8ERS, 8AZI, 8AZL, 8AZM, 8AZN, 8AZO, 8AZP, 8AZC, 8AZD, 7YGQ, 5SOR, 5SOS, 5SOW, 5SOX, 5SP0, 5SP4, 5SP6, 5SP7, 5SPF, 5SQ1, 5SQ6, 5SQ7, 5SQ8, 5SOJ, 5SOK, 5SOL, 5SOM, 5SON, 5SOO, 5SOP, 5SOQ, 5SOT, 5SOU, 5SOV, 5SOY, 5SOZ, 5SP1, 5SP3, 5SP8, 5SP9, 5SPA, 5SPB, 5SPC, 5SPD, 5SPE, 5SPG, 5SPH, 5SPI, 5SPJ, 5SPK, 5SPL, 5SPM, 5SPN, 5SPO, 5SPP, 5SPQ, 5SPR, 5SPS, 5SPT, 5SPU, 5SPV, 5SPW, 5SPX, 5SPY, 5SPZ, 5SQ0, 5SQ2, 5SQ3, 5SQ4, 5SQ5, 5SQ9, 5SQA, 5SQB, 5SQC, 5SQD, 5SQE, 5SQF, 5SQG, 5SQH, 5SQI, 5SQJ, 5SQK, 5SQL, 5SQM, 5SQN, 5SQO, 5SQP, 5SQQ, 5SQR, 5SQS, 5SQT, 5SQU, 5SQV, 5SQW, 5SQX, 5SQY, 5SQZ, 5SR0, 5SR1, 5SR2, 5SR3, 5SR4, 5SR5, 5SR6, 5SR7, 5SR8, 5SR9, 5SRA, 5SRB, 5SRD, 5SRE, 5SRF, 5SRG, 5SRI, 5SRJ, 5SRK, 5SRL, 5SRM, 5SRQ, 5SRR, 5SRT, 5SRU, 5SRV, 5SRW, 5SRX, 5SRY, 5SS0, 5SS1, 5SS2, 5SS3, 5SS4, 5SS5, 5SS6, 5SS7, 5SS8, 5SS9, 5SSA, 5SSB, 5SSC, 5SSD, 5SSE, 5SSF, 5SSG, 5SSH, 5SSI, 5SSJ, 5SSK, 5SSL, 5SSN, 5SSQ, 5SSR, 5SRC, 5SRO, 5SRS, 5SRZ, 5SSM, 5SSO, 5SSP, 7ZQW, 7XC4, 7XAX, 7TX5, 7TWT, 7TWX, 7TWY, 7TX0, 7TX1, 7TWV, 7TWW, 7WQI, 7QG7
**AmpC**	7RU0, 7RTF, 7RTJ, 7FF9, 7FF0, 7FF8, 7FEW, 7E3W, 7RHG, 7NP4, 7LZ4, 7CIN, 6M5P, 6M5Q, 7DOO, 6LOQ, 6RSX, 6PWM, 6PWL, 6XFS, 6YPD, 6YEO, 6YEN, 6KSV, 6TZF, 6TZG, 6TZI, 6TZH, 6TZJ, 6WIF, 6LC8, 6LC9, 6PB4, 6PB5, 6PB6, 6UR3, 6UQS, 6UQT, 6UQU, 6KBY, 6KA5, 6K9T, 6S1S, 5ZYB, 5ZA2	-
**σ2**	7M93, 7M94, 7M95, 7M96, 7MFI	-
**D4**	6IQL	-
