# Supplementary material for: Large scale prospective evaluation of co-folding across 557 Mac1-ligand complexes and three virtual screens
Source: bioRxiv. 2025 Dec 29:2025.12.25.696505. Preprint. [Version 2] doi: 10.64898/2025.12.25.696505 (PMC12776374; doi:10.64898/2025.12.25.696505)
Supplement: Supplement 6 [file NIHPP2025.12.25.696505v2-supplement-6.pdf]

bioRxiv preprint doi: https://doi.org/10.64898/2025.12.25.696505; this version posted December 29, 2025. The copyright holder for this preprint (which was not certified by peer review) is the author/funder, who has granted bioRxiv a license to display the preprint in perpetuity. It is made available under aCC-BY 4.0 International license.

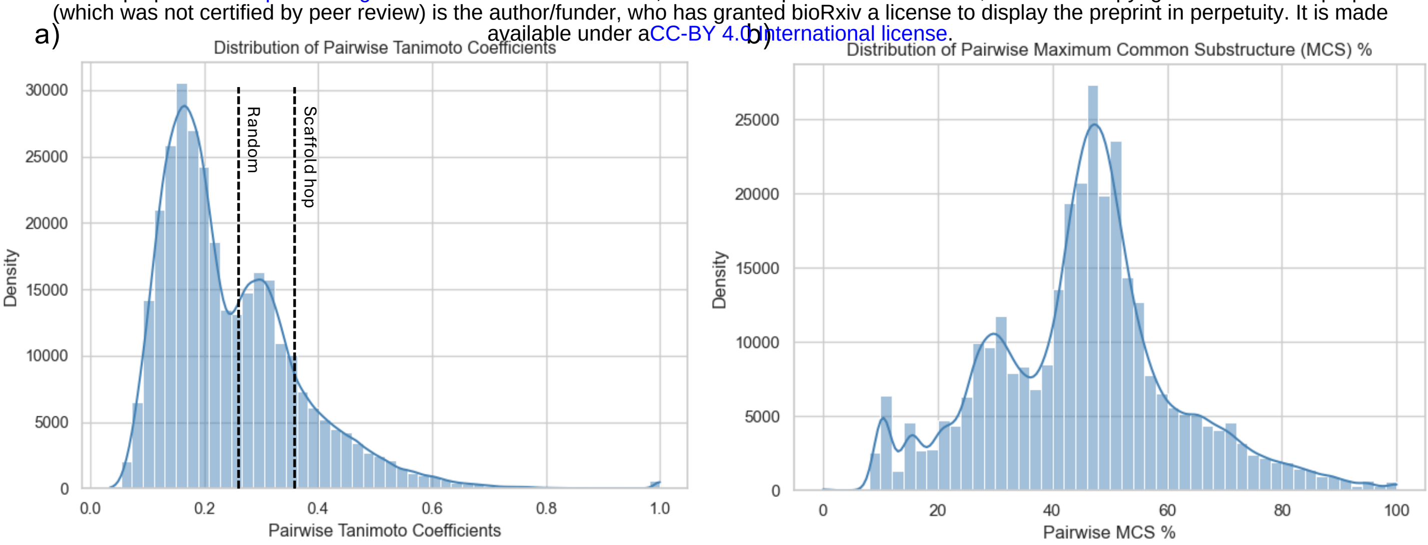

<u>Supplementary Figure 1</u>: **<u>Distribution of pairwise similarity for Mac1 ligands</u>** using a) ECFP4 Tanimoto Coefficients (TC), with lines indicating values for a “scaffold hop” or random distributions. b) Maximum Common Substructure (MCS) similarity values.

bioRxiv preprint doi: https://doi.org/10.64898/2025.12.25.696505; this version posted December 29, 2025. The copyright holder for this preprint (which was not certified by peer review) is the author/funder, who has granted bioRxiv a license to display the preprint in perpetuity. It is made available under aCC-BY 4.0 International license.

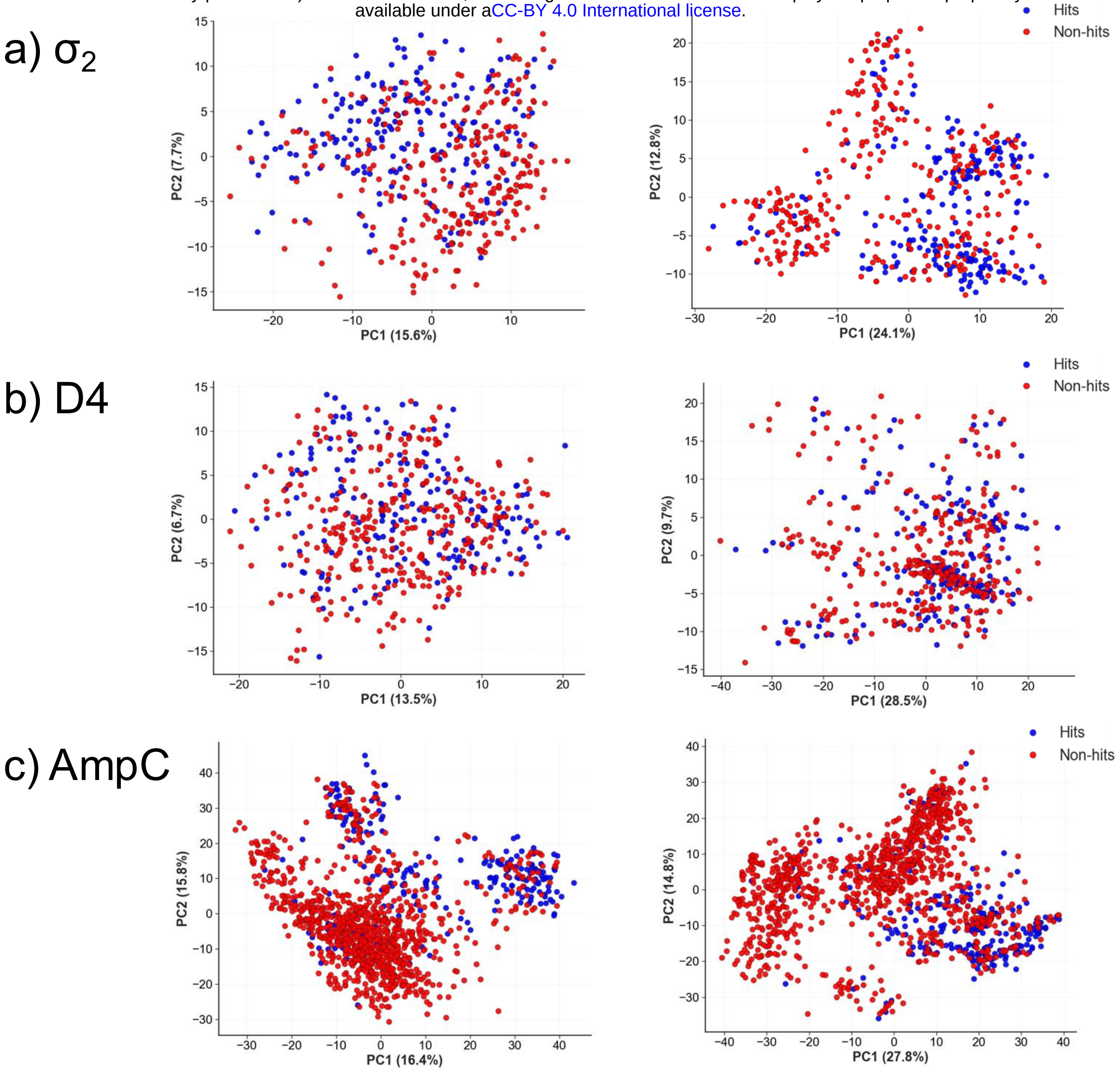

Target proteins	# known ligands	# total	# cluster heads by TC > 0.35	# cluster heads by MCS > 35%
**σ_2**	201	506	348	46
**D4**	205	541	410	47
**AmpC**	247	1,293	500	62
**Mac1***	557	557	30	4

Supplementary Figure 2: Benchmarked datasets are clustered based on TC and MCS% to predict the number of scaffold series. Principal Component Analysis (PCA) plots make pairwise comparisons of molecules in the docked hit lists of a) σ_2, b) D4, c) AmpC for similarity using two different metrics: TC (first column) and MCS% (second column). Red points indicate docked false positives in the hit list, and blue points indicate known actives. Mac1 clustering plots were shown in Fig.1 and an asterisk indicates we only have known ligands for the Mac1 set. The number of scaffolds based on cluster heads are determined by either TC > 0.35, or MCS > 35%. The table gives the summary of the clustering analysis of the datasets used.

bioRxiv preprint doi: https://doi.org/10.64898/2025.12.25.696505; this version posted December 29, 2025. The copyright holder for this preprint (which was not certified by peer review) is the author/funder, who has granted bioRxiv a license to display the preprint in perpetuity. It is made available under aCC-BY 4.0 International license.

a) Examples of mis-predicted alternate conformations (AF3)

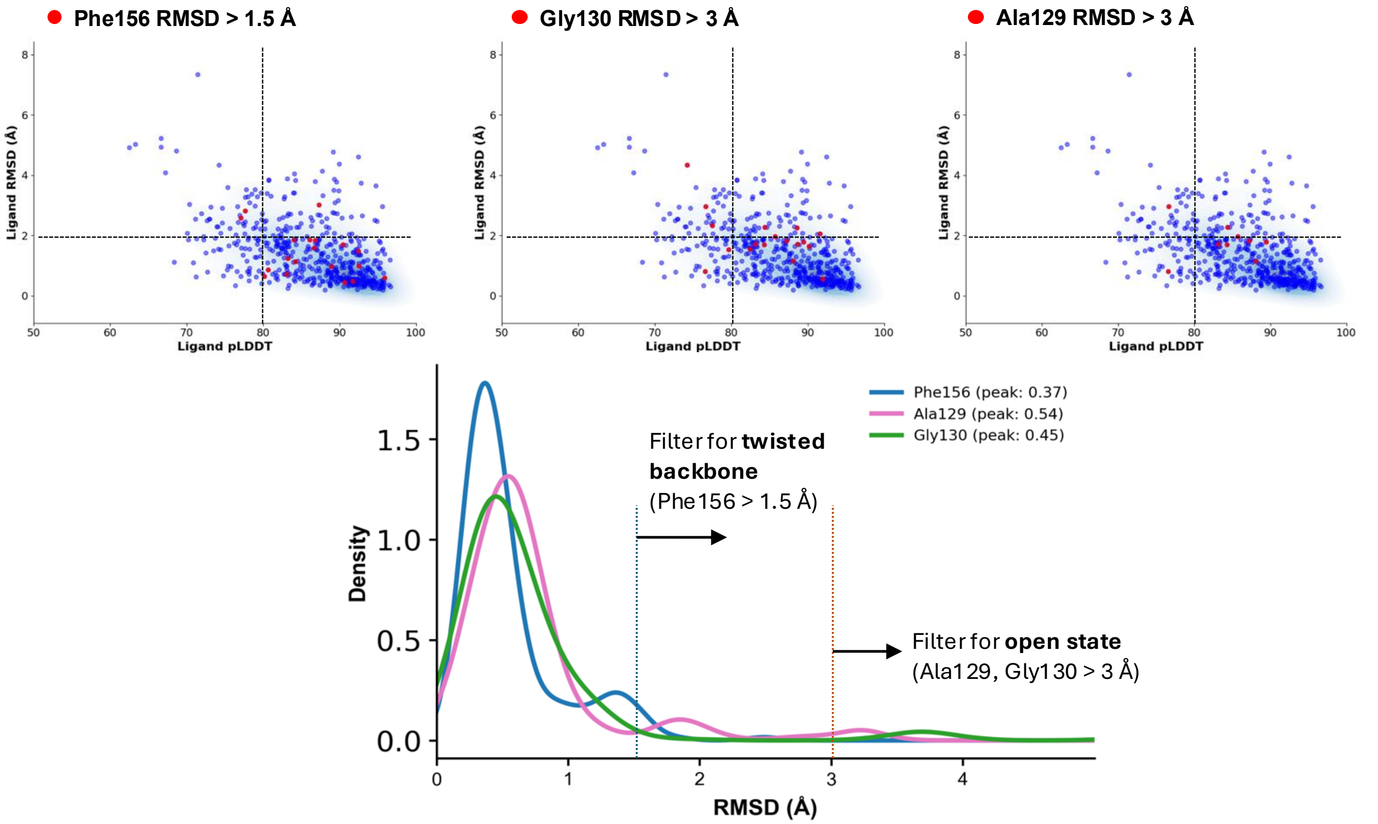

b) Interaction fingerprints across co-folded Mac1 compounds

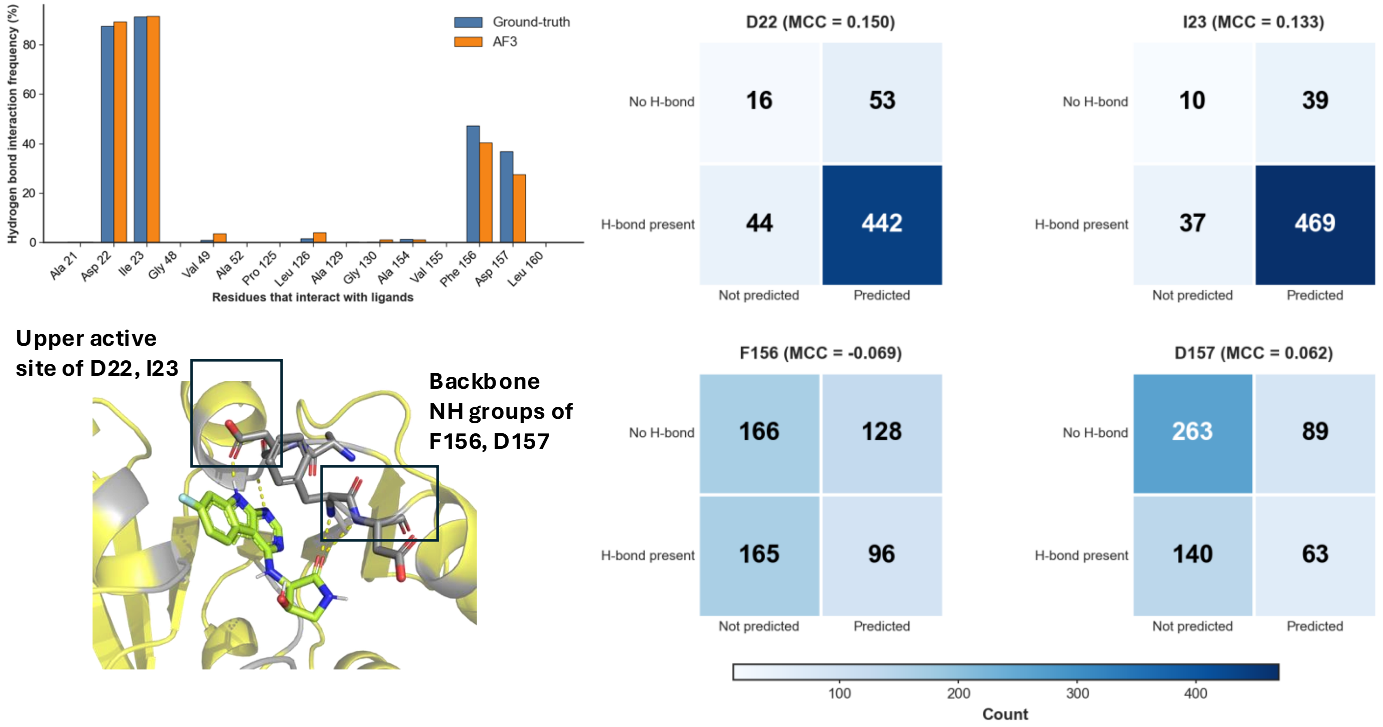

Supplementary Figure 3: Detailed predictions of alternate residue conformations and interactions in co-folded poses. a) Mis-prediction of alternate conformations of the binding pocket, including twisted backbone (Phe156 RMSD > 1.5 Å) and open structure (Gly130 RMSD, Ala129 RMSD > 3 Å) did not produce false positive or false negative pose prediction. The density distribution plot shows RMSD between residues in PDB ID: 5SQW, and 557 complexes obtained from crystallography, a filter that was used to define alternate conformations. b) Hydrogen bonds between ligands and residues within 5 Å were counted for crystal, and for AF3 co-folded structures. Interactions shown with 4 hotspot residues (D22, I23, F156, D157) were compared and confusion matrices show absolute counts of True positives, True negatives, False positives and False negatives. Matthews Correlation Coefficients are calculated for each residue.

bioRxiv preprint doi: https://doi.org/10.64898/2025.12.25.696505; this version posted December 29, 2025. The copyright holder for this preprint (which was not certified by peer review) is the author/funder, who has granted bioRxiv a license to display the preprint in perpetuity. It is made available under aCC-BY 4.0 International license.

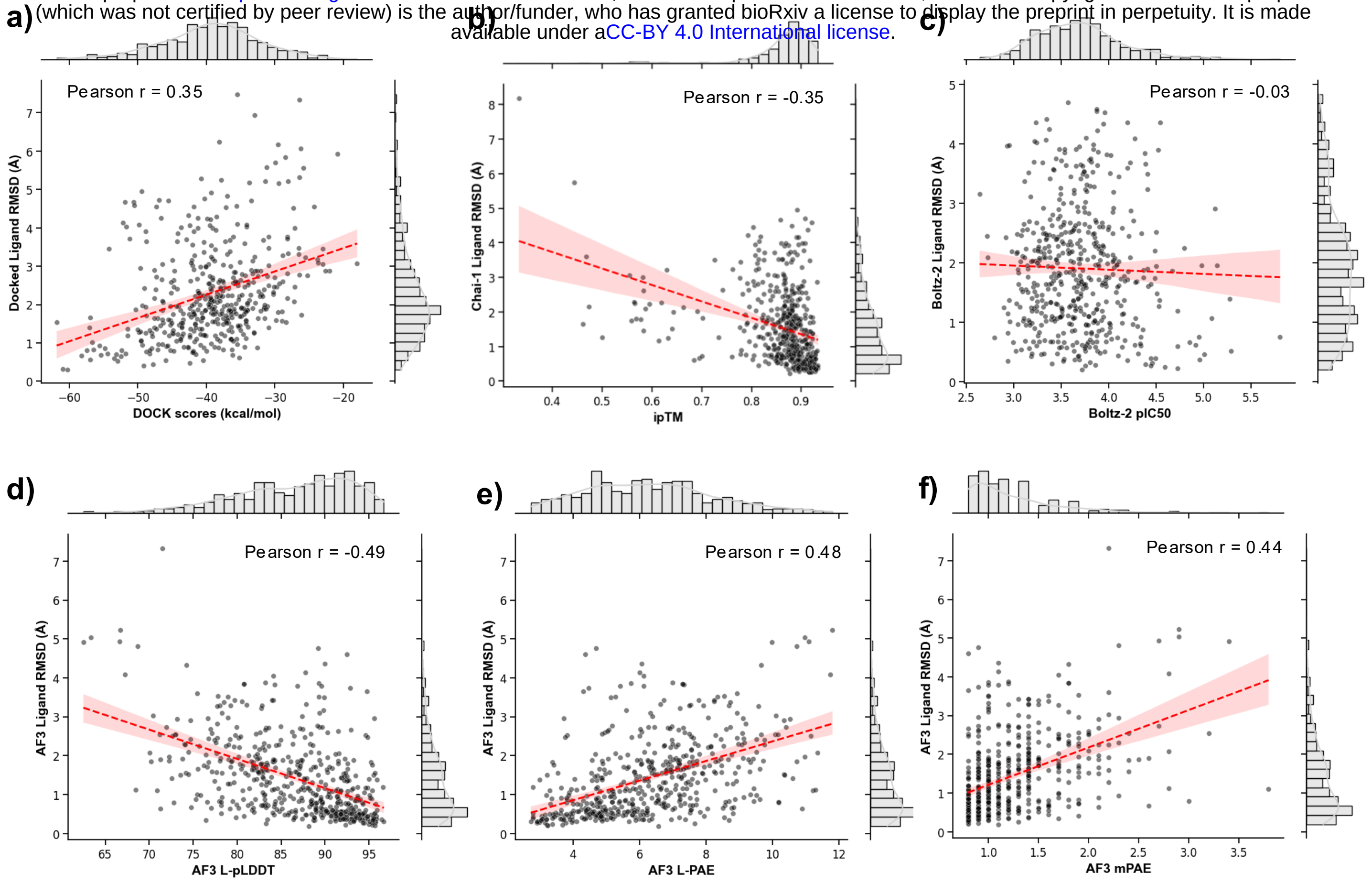

Supplementary Figure 4: Correlations between Mac1 pose recovery with co-folding/docking scoring metrics. a) Docked pose RMSD is compared with DOCK scores (in kcal/mol), b) Chai-1 RMSD is compared with interface predictive TM-Score (ipTM), and c) Boltz-2 pose RMSD is compared with predicted Boltz-2 pIC50 affinity score. AF3 RMSD is compared with three possible scoring metrics from AF3: d) Ligand-specific pLDDT (L-pLDDT), e) Ligand-specific PAE (L-PAE), f) minimum PAE (mPAE).

bioRxiv preprint doi: https://doi.org/10.64898/2025.12.25.696505; this version posted December 29, 2025. The copyright holder for this preprint (which was not certified by peer review) is the author/funder, who has granted bioRxiv a license to display the preprint in perpetuity. It is made available under aCC-BY 4.0 International license.

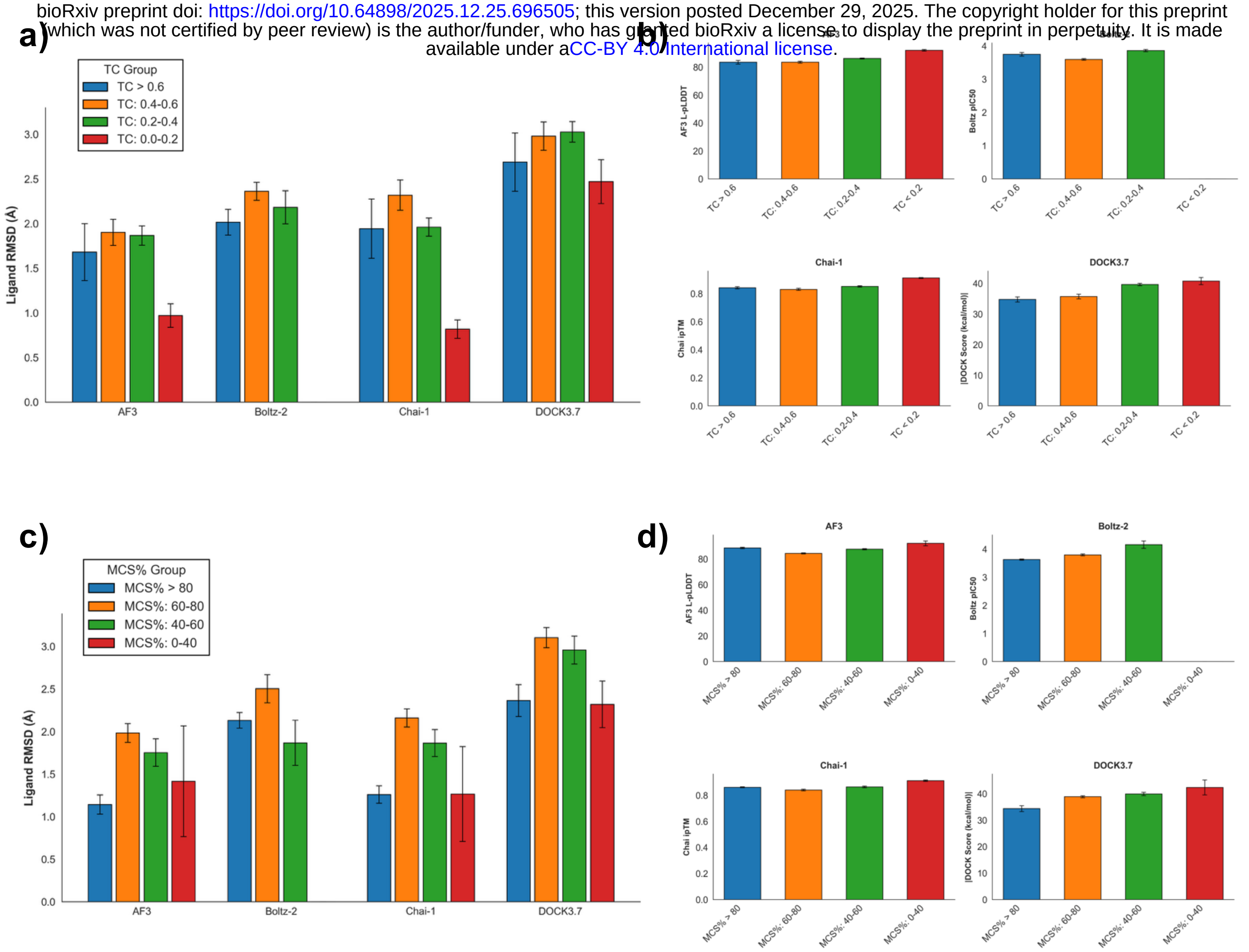

<u>Supplementary Figure 5: Pose accuracy and co-folding docking scores are compared for different similarity bins.</u> a) Ligand RMSD between co-folded poses and docked poses are compared to the ground-truth for different Tanimoto Coefficient (TC) bins (< 0.2, 0.2-0.4, 0.4-0.6, > 0.6), b) Scores for each method (AF3 L-pLDDT, Chai-1 ipTM, Boltz-2 pIC_{50}, DOCK3.7 energies) are compared for different TC bins. c) Ligand RMSD for different Maximum Common substructure (MCS%) bins (< 40, 40-60, 60-80, > 80), d) Scores for each method are compared for different MCS% bins. TC and MCS% of new molecules to the trained set were calculated for each model, and for DOCK3.7 (the only non-co-folding method), the similarity was calculated against the AF3 trained set.

bioRxiv preprint doi: https://doi.org/10.64898/2025.12.25.696505; this version posted December 29, 2025. The copyright holder for this preprint (which was not certified by peer review) is the author/funder, who has granted bioRxiv a license to display the preprint in perpetuity. It is made available under aCC-BY 4.0 International license.

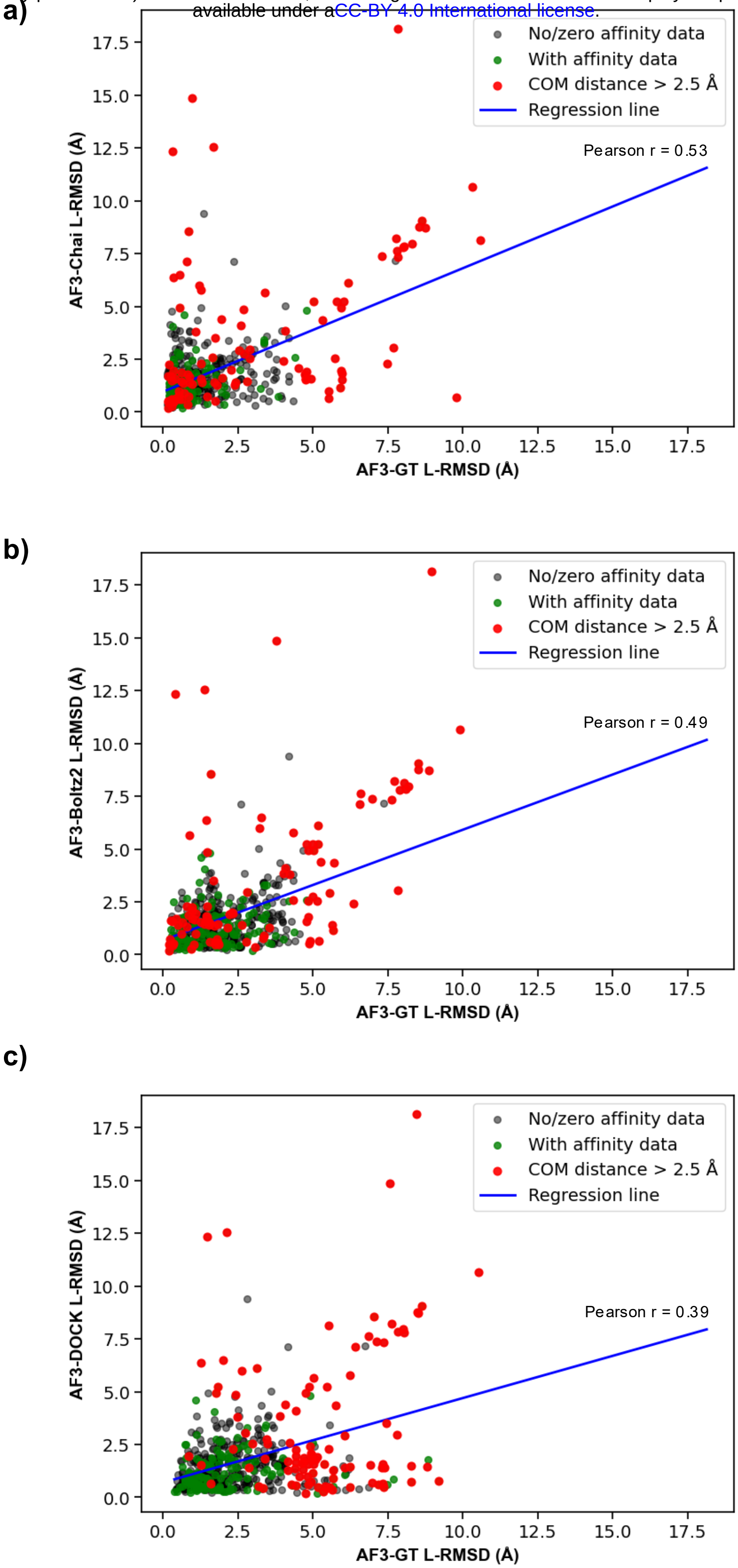

Supplementary Figure 6: **Correlation between AF3 pose-prediction error and errors from other co-folding and docking methods for 557 Mac1 compounds.** a) AF3-Ground truth (GT) L-RMSD vs AF3-Chai L-RMSD, b) AF3-Ground truth L-RMSD vs AF3-Boltz 2 L-RMSD, c) AF3-Ground truth L-RMSD vs AF3-DOCK L-RMSD. Ligands with affinity data (n= 202) are marked in green, those with high COM distance are marked in red, and molecules with no affinity data are marked as grey. Pearson correlation coefficients and regression lines are shown.

bioRxiv preprint doi: https://doi.org/10.64898/2025.12.25.696505; this version posted December 29, 2025. The copyright holder for this preprint (which was not certified by peer review) is the author/funder, who has granted bioRxiv a license to display the preprint in perpetuity. It is made available under aCC-BY 4.0 International license.

Pose recovery by methods

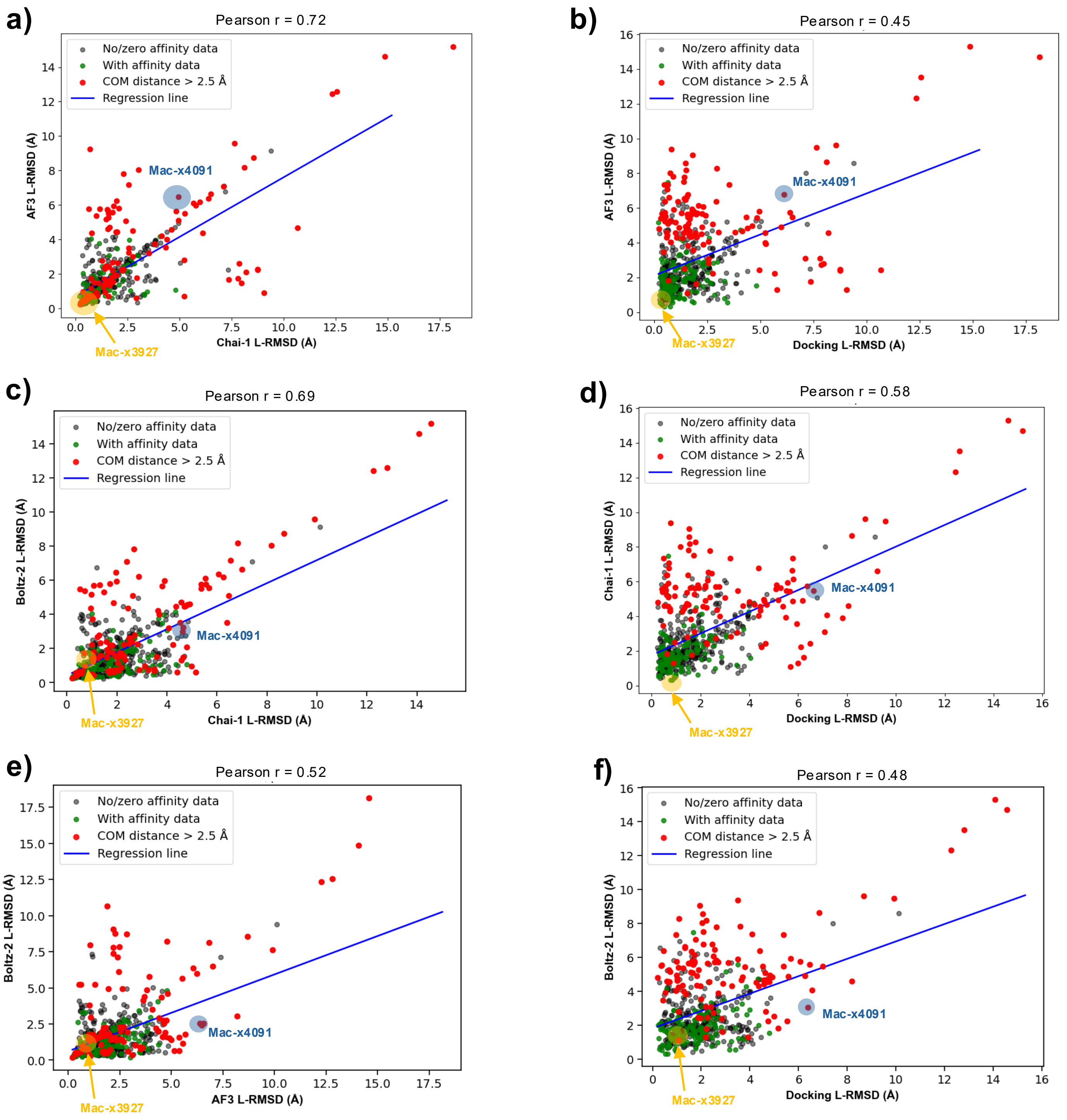

<u>Supplementary Figure 7: Pose recovery by methods of co-folding and docking against the ground-truth pose.</u> a) AF3 vs. Chai-1, b) AF3 vs. DOCK3.7, c) Boltz-2 vs. Chai-1, d) Chai-1 vs. DOCK3.7, e) Boltz-2 vs. AF3, f) Boltz-2 vs. DOCK3.7. Molecules indicated (Mac-x4091, Mac-x3927) are exemplary ligands highlighted in **<u>Fig. 3</u>**, and L-RMSD values quoted here are all compared against the ground-truth pose.

bioRxiv preprint doi: https://doi.org/10.64898/2025.12.25.696505; this version posted December 29, 2025. The copyright holder for this preprint (which was not certified by peer review) is the author/funder, who has granted bioRxiv a license to display the preprint in perpetuity. It is made available under aCC-BY 4.0 International license.

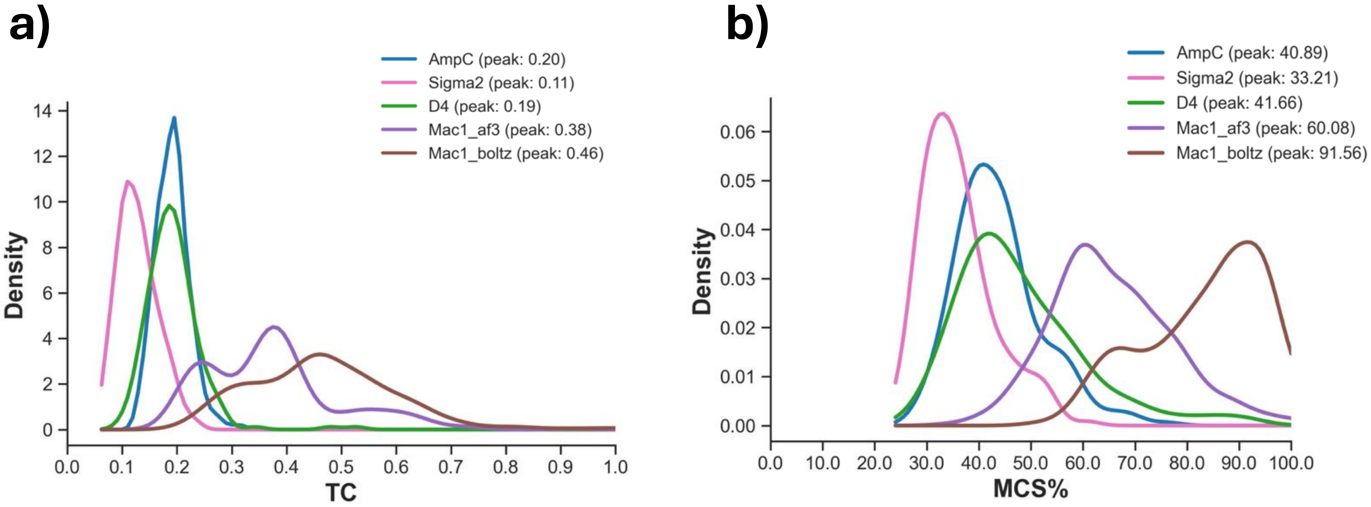

<u>Supplementary Figure 8: Actives from the benchmarked datasets are compared with resolved structures used to train AF3, Chai-1 and Boltz-2.</u> Actives from AmpC (n = 247), D4 (n = 205), σ_2 (n = 201), Mac1 (n = 557) were compared with corresponding trained sets, to give an indication how similar the actives were to those trained by co-folding models, based on a) Tanimoto coefficients, and b) Maximum Common Substructures (%). AF3 and Chai-1 have similar cutoff dates (indicated as Mac1_af3), but Boltz-2 has been trained with more recent PDB and is labelled as Mac1_boltz. Exact PDB IDs used for each model system is shown in Supplementary Table 6.

bioRxiv preprint doi: https://doi.org/10.64898/2025.12.25.696505; this version posted December 29, 2025. The copyright holder for this preprint (which was not certified by peer review) is the author/funder, who has granted bioRxiv a license to display the preprint in perpetuity. It is made available under aCC-BY 4.0 International license.

a) σ_2 (Baseline MAE = 0.640 kcal)

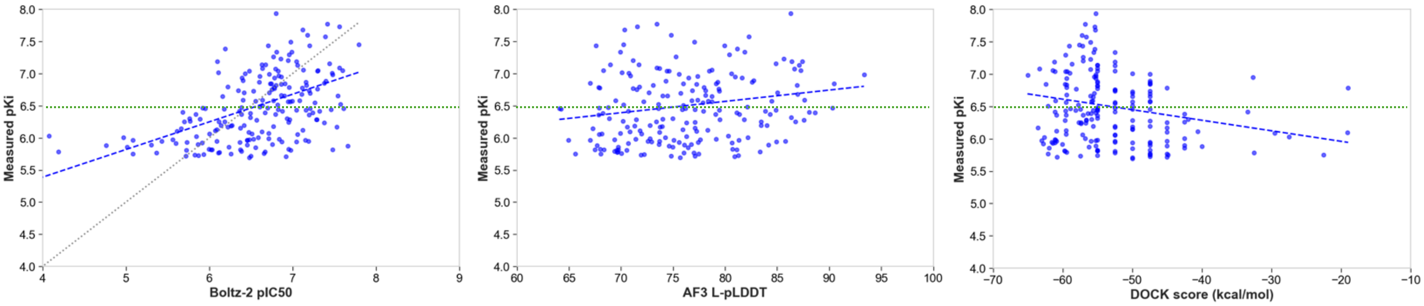

Pearson r = 0.486
MAE (y = x) = 0.694 kcal
MAE (regression line) = 0.547 kcal

Pearson r = 0.183
MAE (regression line) = 0.637 kcal

Pearson r = -0.211
MAE (regression line) = 0.634 kcal

b) D4 (Baseline MAE = 0.578 kcal)

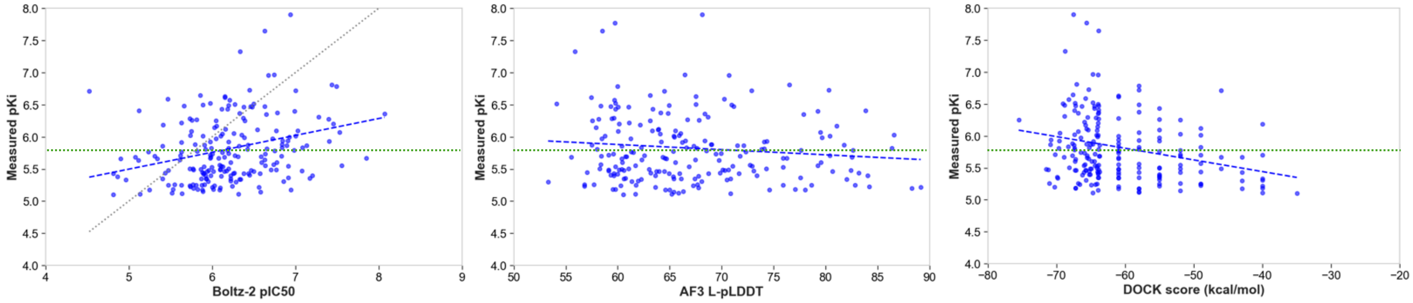

r = 0.284
MAE (y = x) = 0.888 kcal
MAE (regression line) = 0.554 kcal

r = -0.109
MAE (regression line) = 0.591 kcal

r = -0.247
MAE (regression line) = 0.572 kcal

c) AmpC (Baseline MAE = 0.424 kcal)

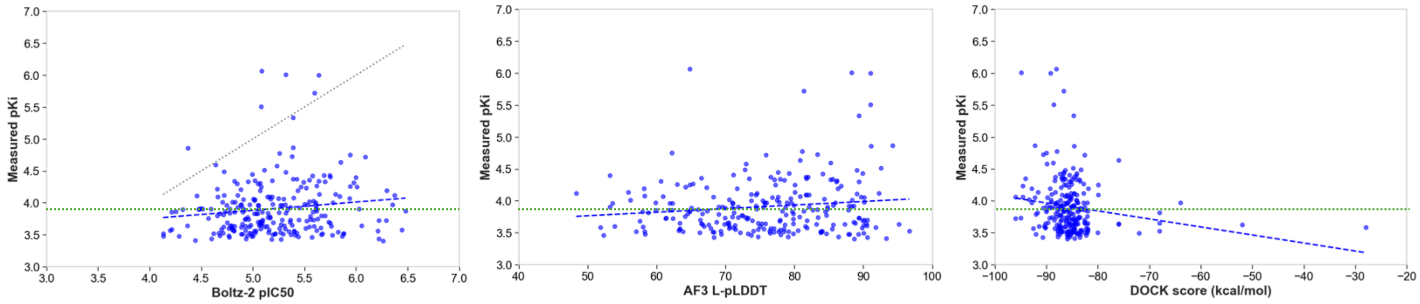

r = 0.139
MAE (y = x) = 1.805 kcal
MAE (regression line) = 0.320 kcal

r = 0.131
MAE (regression line) = 0.423 kcal

r = -0.169
MAE (regression line) = 0.419 kcal

**Bonferroni-corrected p-values**	**Sigma2**	**AmpC**	**D4**
MAE_AF3 vs MAE_Boltz-2	0.0722	1.00	1.00
MAE_AF3 vs MAE_DOCK	1.00	1.00	1.00
MAE_Boltz-2 vs MAE_DOCK	0.0674	1,00	1.00
**Friedman χ^2**	**18.79 (p = 8.31e-05)**	**0.073 (p = 0.964)**	**0.48 (p = 0.786)**

Supplementary Figure 9: Discriminative power of co-folding and docking scores by correlation against experimentally measured pK_i across different targets: a) σ_2 (n = 201 actives), b) D4 (n = 205 actives), c) AmpC (n = 247 actives). The leftmost panel is for Boltz-2 pIC_{50} affinities, the middle panel for AF3 L-pLDDT and the rightmost panel for DOCK scores. All these scores are compared against measured pK_i values. Mean Absolute Errors and Pearson correlation coefficients are calculated. Blue lines show the corrected regression line, black dotted line before the correction, and green horizontal line shows the baseline at an average measured pKi value. Friedman test with Conover post-hoc comparisons and Bonferroni corrections compare MAE between methods for each target system.

bioRxiv preprint doi: https://doi.org/10.64898/2025.12.25.696505; this version posted December 29, 2025. The copyright holder for this preprint (which was not certified by peer review) is the author/funder, who has granted bioRxiv a license to display the preprint in perpetuity. It is made available under aCC-BY 4.0 International license.

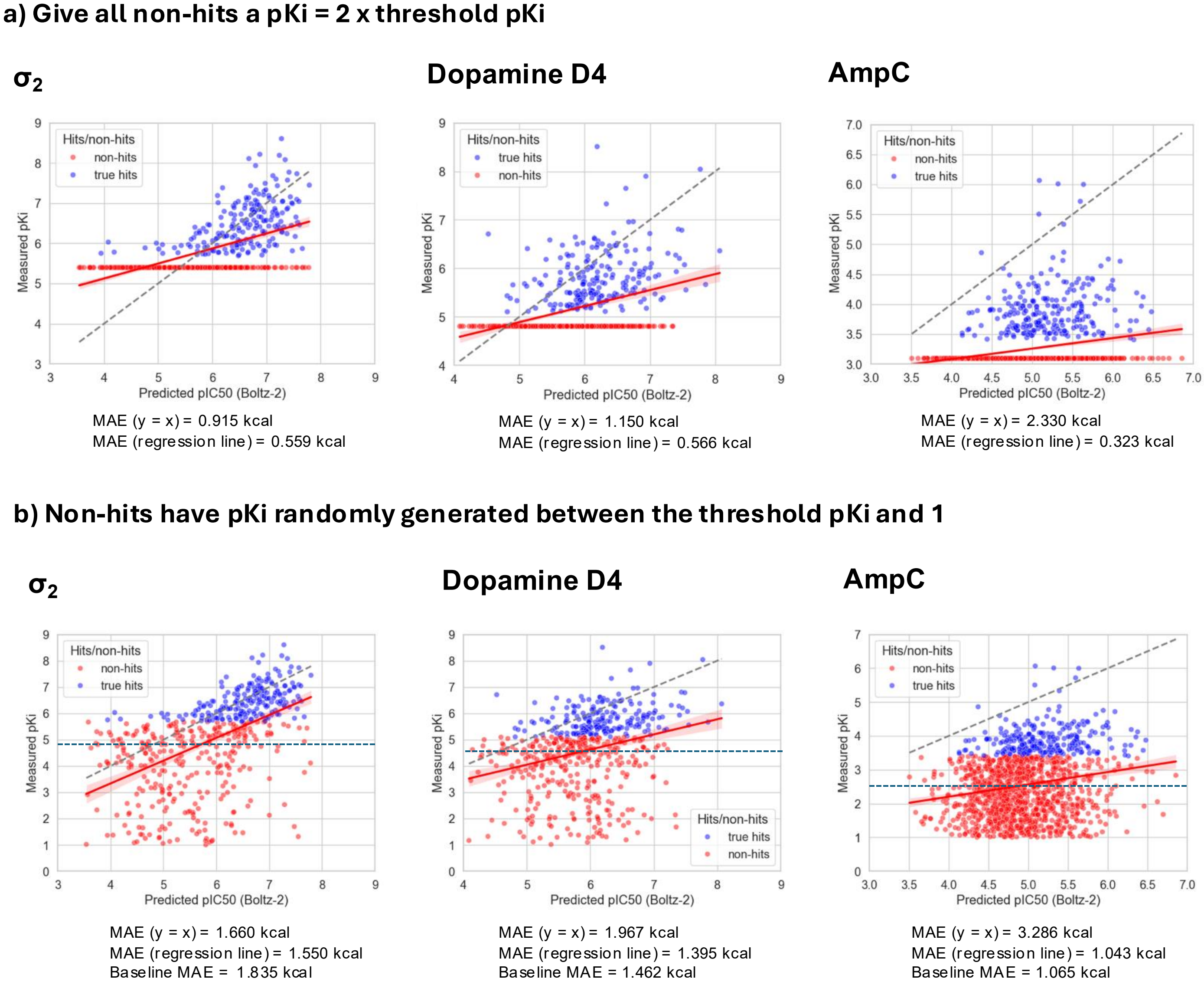

<u>Supplementary Figure 10: Different methods of treating randomly assigned pK_i values for docked false positives in σ_2, D4, AmpC docked lists</u>. Mean absolute errors (before linear correction is shown in grey dotted line, after linear correction is the red regression line and blue line indicates the baseline when we predict all values at measured pK_i) between the measured pK_i and Boltz-2 pIC_{50} affinity scores when a) all non-hits are assigned a pK_i = 2 x $pK_{i,threshold}$; b) non-hits are randomly assigned a pK_i value between the $pK_{i,threshold}$ and 1. Baseline MAE is only quoted for b), since the error could be misinterpreted when all non-binders' pK_i values are fixed.

bioRxiv preprint doi: https://doi.org/10.64898/2025.12.25.696505; this version posted December 29, 2025. The copyright holder for this preprint (which was not certified by peer review) is the author/funder, who has granted bioRxiv a license to display the preprint in perpetuity. It is made available under aCC-BY 4.0 International license.

a) Sigma2

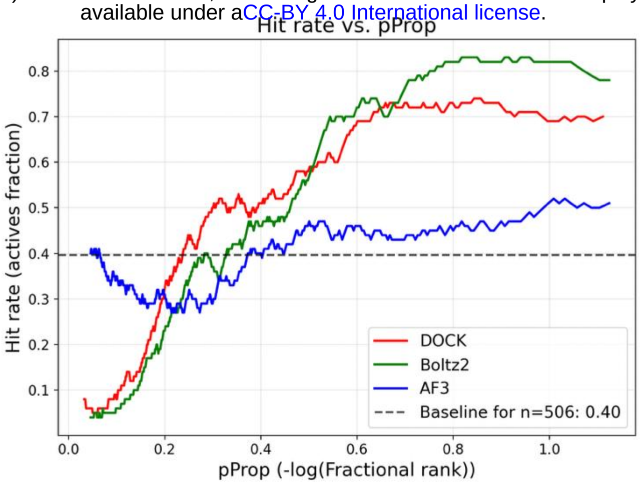

b) D4

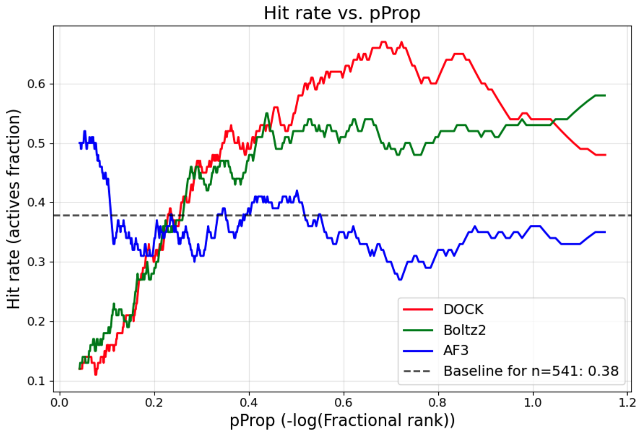

c) AmpC

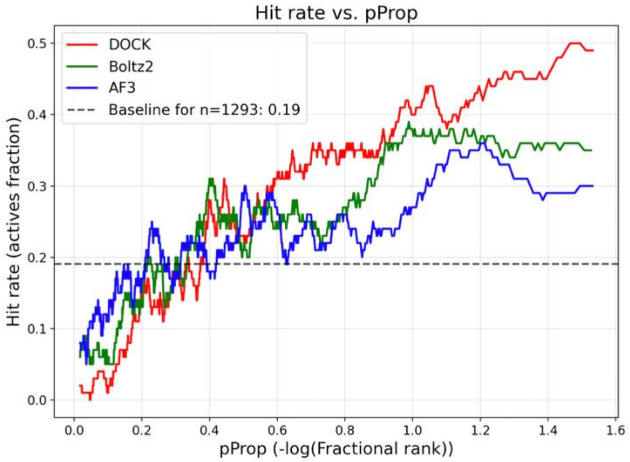

Supplementary Figure 11: Hit rate curves for co-folding and docking scores for the three experimental benchmark datasets. Hit rate curves are plotted over a rolling window (window = 100 for AmpC, σ_2 and 50 for D4) after ranking with pProp from the docked hit lists, from three different targets: a) σ_2 (201 actives, 305 non-binders), b) Dopamine D4 (205 actives, 336 non-binders), c) AmpC β-lactamase (247 actives, 1,046 non-binders).

bioRxiv preprint doi: https://doi.org/10.64898/2025.12.25.696505; this version posted December 29, 2025. The copyright holder for this preprint (which was not certified by peer review) is the author/funder, who has granted bioRxiv a license to display the preprint in perpetuity. It is made available under aCC-BY 4.0 International license.

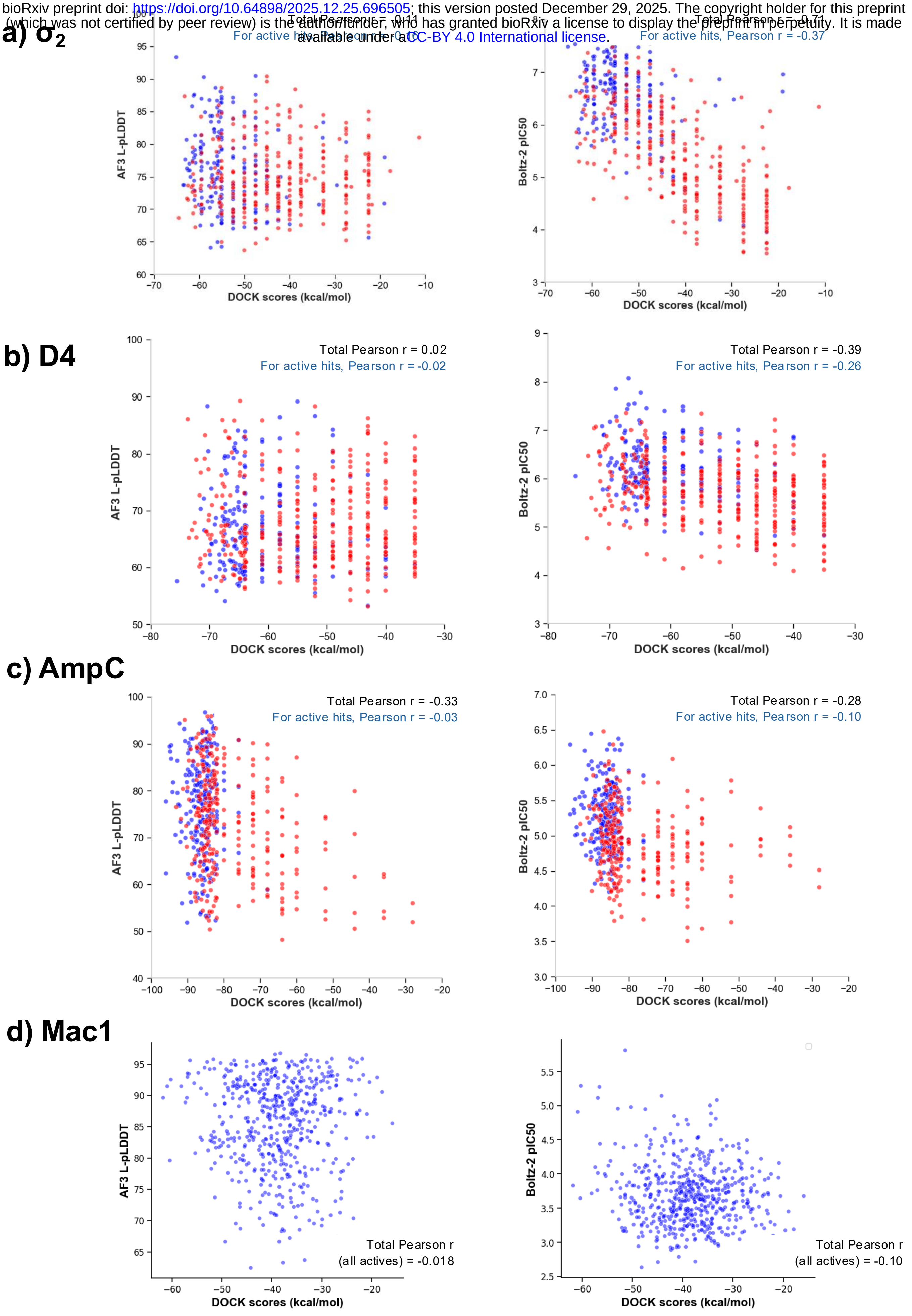

Supplementary Figure 12: Correlation between DOCK scores and some co-folding scores (AF3 L-pLDDT, Boltz-2 pIC50). Blue points indicate known actives and red points are non-binders for each target system: a) σ_2, b) D4, c) AmpC and d) Mac1. Note that Mac1 dataset comprises only actives.
